# Atrial Fibrillation in Chronic Kidney Disease: An Overview

**DOI:** 10.7759/cureus.27753

**Published:** 2022-08-07

**Authors:** Sai Gadde, Revanth Kalluru, Swathi Priya Cherukuri, Rahul Chikatimalla, Thejaswi Dasaradhan, Jancy Koneti

**Affiliations:** 1 Research, Kamineni Institute of Medical Sciences, Narketpally, IND; 2 Research, Narayana Medical College, Nellore, IND

**Keywords:** thromboembolic stroke, inflammatory bio markers, cardiac remodelling, anti-arrhythmic medications, anti-coagulant therapy, chronic kidney disease (ckd), atrial fibrillation (af)

## Abstract

Chronic kidney disease (CKD) is a condition that can be caused due to any etiology leading to structural damage to the kidney, which can be measured by a decrease in estimated glomerular filtration rate (eGFR) and the presence of damage biomarkers for more than three months. This article has discussed the causal relationship between atrial fibrillation (AF) and CKD, a few of them being inflammation, renin-angiotensin-aldosterone system (RAAS) activation, anemia, and uremia associated with CKD. This review mentioned the clinical impact of the presence of AF in CKD patients. The presence of AF in CKD patients aggravates the renal dysfunction, which in turn adds to the generation of AF. This article explores the various pharmacological and interventional treatment modalities, including antiarrhythmics, anticoagulants, and cardiac ablation, and their complications, leading to restricted usage in CKD patients.

## Introduction and background

Chronic kidney disease (CKD) is a declined state of kidney function where the estimated glomerular filtration rate (eGFR) is <60 mL/min/1.73 m^2^ and/or the presence of kidney damage markers for at least three months. The commonly used markers of kidney damage are urine albumin and serum creatinine [1]. CKD is classified into five stages based on the eGFR, and it is elaborated in Table 1 below [2].

**Table 1:** Chronic kidney disease (CKD) classification. The table is adapted from Stevens and Levin (2013) [2].

Stage	Estimated glomerular filtration rate value in mL/min/1.73 m^2^	Classification
I	≥90	Normal or high
II	60-89	Mildly decreased
IIIa	45-59	Mild-to-moderately decreased
IIIb	30-44	Moderate-to-severely decreased
IV	15-29	Severely decreased
V	<15	Kidney failure

Richard Bright, an English physician, was the first to publish his findings on kidney diseases in 1827. He was the first to correlate abnormal renal anatomy and urine findings (albuminuria) to clinical signs. Kidney disease was called Bright's disease for almost a century and is still familiar to this day [3]. The worldwide prevalence of CKD is estimated to be 8-16% [4]. The prevalence climbs up to 23.4-35.8% in patients aged over 64 years, which suggests that increasing age contributes to increasing CKD [5]. The prevalence of CKD is more in women (12.6%) compared to men (9.7%). CKD is equally prevalent in Caucasians (11.6%) and African Americans (11.2%) adults [6,7]. It costs over $60 billion annually to take care of CKD and end-stage renal disease (ESRD) patients aged over 65 years, which accounts for 24% of the total medicare expenses in 2011 in the United States of America (USA) [8].

The leading risk factors for CKD in many countries, both developed and developing, are diabetes and hypertension. In contrast, conditions like glomerulonephritis and some unknown causes are more common in Sub-Saharan Africa and Asian countries [9]. Patients with CKD present with symptoms of foamy urine (a sign of albuminuria), nocturia, hematuria (blood in urine), flank pain, and reduced urine output. Patients with advanced stages of CKD may complain of decreased appetite, fatigue, nausea, dyspnea, vomiting, mental status alteration, and peripheral edema [10]. The laboratory evaluations done for CKD screening are serum creatinine to estimate eGFR, serum electrolytes (sodium, potassium, bicarbonate, chloride), urine pH, albumin-creatinine ratio in a random untimed urine sample, and imaging of kidneys [11]. Early-stage CKD treatment focuses on comorbid illnesses such as diabetes, hypertension, and cardiovascular disease, in order to lower the risk of complications and progression of CKD. Antihypertensive drugs (especially angiotensin-converting enzyme inhibitors and angiotensin II-receptor blockers), lipid-lowering therapies, and dietary changes are also options for treatment [12]. Atrial fibrillation (AF) is a cardiac arrhythmia common in CKD, complications of AF range from harmless palpitations to fatal events such as stroke and death. The management of AF can be quite challenging in patients with CKD, which requires continuous cardiac monitoring, and medical therapy should take into account the complications of thromboembolic events and bleeding [13,14]. This review article aimed to proffer a new perspective on the correlation between AF and CKD by highlighting the pathophysiology of AF in patients with CKD and underlining the clinical impact and therapeutic possibilities.

## Review

Pathogenesis of AF in CKD

Inflammation

**Figure 1:**
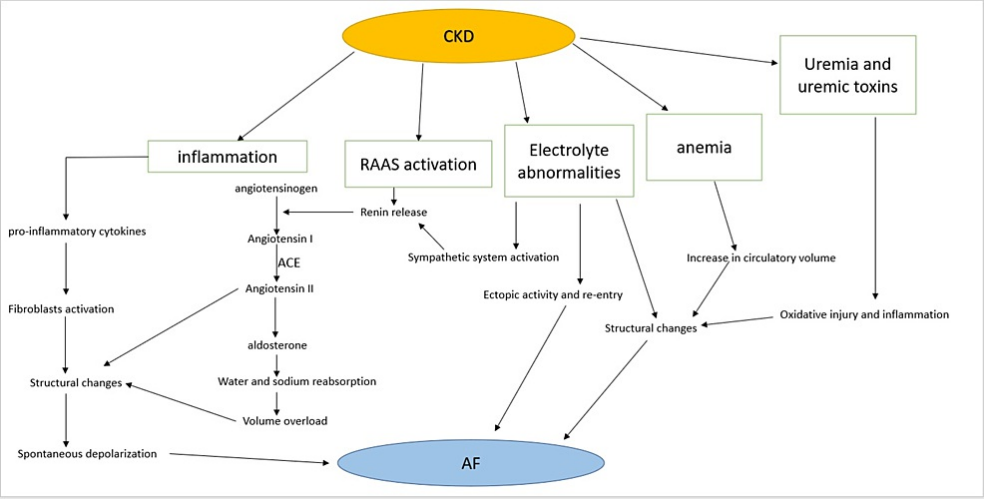
Pathogenesis of AF in CKD patients. CKD: chronic kidney disease; AF: atrial fibrillation; RAAS: renin-angiotensin-aldosterone system; ACE: angiotensin-converting enzyme The image is created by the author (Sai Varun Gadde) of this study.

The occurrence of AF in patients with CKD is multifaceted, one of the causative factors is inflammation which is linked with CKD (Figure 1). CKD patients often exhibit a chronic increase in inflammatory markers like c-reactive protein (CRP), interleukin-6 (IL-6), fibrinogen, and a few other molecules; as the disease progresses the severity increases [15,16].

Inflammasomes are high-molecular-weight, multiprotein complexes present in the cytoplasm of immune cells and can also be found in non-immune cells like renal tubular cells as well as podocytes [17-19]. They are a part of the innate immune system, which identifies molecules called pathogen-associated molecular patterns (PAMPs) and danger/damage-associated molecular patterns (DAMPs) released in response to stress, tissue injury, and apoptosis with the help of pattern recognition receptors (PRR) [20-22]. There are several different families of PRR, like the transmembrane Toll-like receptors (TLRs) and C-type lectin receptors (CLRs), they recognize extracellular PAMPs and DAMPs. Retinoic acid-inducible gene (RIGs) receptors and intracellular nucleotide-binding and oligomerization domain (NOD)-like receptors (NLRs) recognize intracellular PAMPs and DAMPs [23,24].

In kidney injury of any underlying etiology, there is cell damage which leads to the release of several DAMPs like high-mobility group box 1, heat shock protein 70, biglycan, and hyaluronan synthase 1, which bind to NOD-like receptor P3 (NLRP3) a subtype of NLRs leading to activation of NLRP3 which in turn activates the inflammasome. The NLRP3 inflammasome can also be activated as a result of mitochondrial and endothelial dysfunction in CKD patients with uremia undergoing dialysis [25]. In CKD, the activation of NLRP3 can also occur through the generation of reactive oxygen species (ROS) via nicotinamide adenine dinucleotide phosphate (NADPH) oxidase, which is known as the ROS model [26].

According to the ion flux model, NLRP3 can be activated by potassium, calcium, and proton flux [27]. The lysosomal rupture model states that NLRP3 can be activated by lysosomal proteases like cathepsin B and L, which are released into the cytosol due to the disruption of cellular and lysosomal membrane integrity following an injury [28,29]. Activation of the inflammasome triggers the NF-kB pathway leading to the production of proinflammatory cytokines like IL-6, IL-1, and tumor necrosis factor-alpha (TNF α), which can lead to low-grade systemic inflammation [15,30]. As a result of chronic inflammation, there is an increase in inflammatory markers like CRP, IL-6, fibrinogen, and a few other molecules seen in CKD [31].

Chronic systemic inflammation can cause fibrosis, hypertrophy, and cellular apoptosis in the atria. Cardiac fibroblasts, which cause fibrosis, are activated by cytokines and growth factors which in turn are associated with systemic inflammation [32]. Fibroblasts are non-excitable cells, but they can conduct currents between the cardiac myocytes via connexins. This leads to heterogeneity of current conduction, action potentials shortening, resting cardiac myocytes depolarization, and spontaneous phase 4 depolarization causing AF [33]. A cohort study performed by Amdur et al. in 2016, tried to examine the relationship between inflammation and AF in 3762 adults with CKD. At baseline, the presence of AF was established by self-report and electrocardiogram (ECG), and the plasma concentrations of IL-1, IL-6, TNF α, transforming growth factor-beta, high sensitivity CRP, and fibrinogen were also measured. A total of 642 patients had a history of AF at baseline, and with a mean follow-up of 3.7 years, 108 patients developed new-onset AF. Initially, the association was not so significant, but after adjusting factors like demographic characteristics, co-morbidities, lab values, medication use, and echocardiographic variables, the association between plasma IL-6 and AF was significant. AF at baseline had an odds ratio (OR) of 1.61 (95% confidence interval {CI}, 1.21-2.14; p=0.001) and the new-onset AF had OR of 1.25 (95% CI, 1.02-1.53; p=0.03). This study concluded that inflammatory cytokines like IL-6 are associated with AF in CKD patients (Table 2) [34]. In another case-control study performed by Chung et al. in 2001, CRP was compared between 131 patients with atrial arrhythmia and 71 control patients. CRP was higher in patients with arrhythmia than in controls. The study concluded that inflammatory states with elevated CRP promote AF (Table 2) [35].

**Table 2:** Summarized studies to understand the relation between inflammation and AF. CKD: chronic kidney disease; AF: atrial fibrillation; IL-6: interleukin-6; CRP: c-reactive protein; CI: confidence interval; OR: odds ratio

Reference	Year	Design	Population	Methods/results	Conclusion
Amdur et al. [34]	2016	Cohort study	3762 adults with CKD	Measuring plasma concentrations of IL-6, CRP, and fibrinogen and the plasma IL-6 in CKD patients and AF had an OR 1.61 (95% CI 1.21-2.14) and new-onset AF had an OR 1.2 (95% CI 1.02-1.53)	Inflammation can lead to AF in CKD patients
Chung et al. [35]	2001	Case-control study	131 individuals with atrial arrhythmias and 71 control individuals	Measuring CRP	Inflammatory states with elevated CRP promote AF

Renin-Angiotensin-Aldosterone System, Neurohormonal, and Other Pathways

Sodium and water balance in the human body are maintained by the renin-angiotensin-aldosterone system (RAAS), sympathetic nervous system (SNS), natriuretic peptides (NP), and antidiuretic hormone (ADH), which is also known as arginine vasopressin (AVP) [36]. Increased salt and water excretion or loss led to the activation of RAAS/SNS, which releases renin from the juxtaglomerular epithelioid (JGE) cells. Renin helps convert angiotensinogen to angiotensin I, angiotensin-converting enzyme (ACE) then converts angiotensin I into angiotensin II which aids in the release of aldosterone, reabsorbs sodium and water to help maintain the effective circulating volume (ECV) [37]. There is an increase in the plasma renin concentration as an adaptation to compensate for the loss of nephrons due to kidney damage in CKD [38]. The increase in RAAS from raised renin activity leads to large amounts of sodium and water retention leading to volume overload [36]. Atrial natriuretic peptide (ANP) is secreted in response to atrial stretch as a result of volume and pressure overload, and its effects are mainly on blood vessels, kidneys, and the adrenals to reduce the blood volume. It acts by inhibiting the secretion of renin and aldosterone [39]. ANP increases blood flow to the kidneys by enhancing vasodilation via inhibiting the release of endothelin which is a vasoconstrictor and increasing the concentration of cyclic guanosine monophosphate (cGMP). This causes pressure-induced natriuresis and they also inhibit sodium reabsorption from the epithelial sodium channels [40]. Yet the net effect is an increase in blood volume due to a greater action of aldosterone from RAAS than the ANP, causing sodium and water retention [36].

Anemia is a known complication in CKD patients due to decreased erythropoietin (EPO). Anemia can cause an increase in circulating volume by decreasing afterload and increasing preload as a result of a decrease in oxygen supply to the tissues [41,42]. The volume overload causes dilation of the left ventricle, which can progress to left ventricular hypertrophy (LVH) and heart failure (HF), this may result in an increase in end-diastolic pressure causing left atrial enlargement (Figure 1) [43,44]. These series of events can stretch the atrial wall, predisposing it to the development of AF [45]. Seko et al. in 2018 performed a study in Japan on a hospital-based population to determine the relationship between paroxysmal AF and their left ventricular (LV) remodeling, left atrial (LA) size, and left ventricular ejection fraction (LVEF). They performed a retrospective study on a total of 4444 patients, out of which 419 patients were excluded due to a history of previous myocardial infarction, and a total of 11 others were excluded due to lack of data on body surface area. The remaining 4014 patients were categorized into four groups which are normal geometry, concentric remodeling, concentric hypertrophy, and eccentric hypertrophy. The mean left atrial volume indices of these groups were 22.5, 23.8, 33.3, and 37.0 mm/m^2^, respectively, and the prevalence of AF was 10.4%, 10.5%, 14.8%, and 16.8%, respectively. This concluded that the prevalence of AF is more in patients with increasing left atrial volume due to left ventricular structural changes [46]. The rise in levels of angiotensin II in CKD patients as a consequence of overactive RAAS causes atrial fibrosis, which may predispose them to AF. Angiotensin II stimulates the gene expression of transforming growth factor-beta (TGF-β), collagen, and fibronectin which leads to fibroblast proliferation and an increase in the extracellular matrix (ECM) [33,47,48]. CKD patients may have abnormalities in calcium and phosphate metabolism, which predisposes them to valvular heart diseases like mitral valve or aortic valve calcification, these changes cause an increase in the left atrial pressure leading to AF [45,49]. Calcium abnormalities can also cause ectopic activity and re-entry, which play a role in the pathogenesis of AF (Figure 1) [50].

In CKD patients, the pulmonary vein (PV) cardiomyocytes are calcium overloaded. This leads to faster PV beating rates and may cause ryanodine receptor (RyR) dysfunction prolonging the sarcoplasmic reticulum (SR) calcium release predisposing to AF [51]. AF can also occur in CKD patients undergoing dialysis as a result of electrolytes disturbances like hypokalemia [52]. In CKD patients, there is an accumulation of urea and uremic toxins, such as indoxyl sulfate (IS), p-Cresol (PC), and p-Cresol sulfate (PCS). This may lead to AF as a consequence of cardiovascular remodeling, fibrosis, and oxidative injury through neurohormonal activation, oxidative injury, and inflammation (Figure 1) [53]. Aoki et al. published a study in 2015 to assess the role of indoxyl sulfate (IS) (a uremic toxin) in causing cardiac fibrosis and AF in renal dysfunction. A total of 138 male Sprague-Dawley rats were used and were randomly assigned into 5/6 nephrectomy (5/6 Nx) with the vehicle, 5/6 Nx with AST‐120, sham procedure with the vehicle, and sham procedure with AST‐120. AST-120 and vehicle were administered for four weeks. There was a significant increase in serum IS levels in the 5/6 Nx group. As a result, an increased expression of malondialdehyde which is an indicator of oxidative stress was upregulated in the left atrium of 5/6 Nx rats and increased the expression of NADPH oxidase 2 and 4. This leads to an increase in the expression of profibrotic molecules like TGF-β, α‐smooth muscle actin, and collagen type 1 in the left atrium causing atrial fibrosis; predisposing to AF in electrophysiological experiments. This study concluded that uremic toxins like IS could cause atrial fibrosis and AF in renal dysfunction patients [54].

Clinical impact of AF on CKD

AF is one of the most common cardiac arrhythmias in the general population. Its prevalence is based on age and ranges between 0.4% and 1% and may reach up to 8% in older adults above the age of 80 years [55]. CKD is a disorder in which there is a decline in renal function with or without any structural changes, which contributes to cardiovascular dysfunction. The data from the health system mentioned that about 10% population worldwide is affected, and there is a 29.3% increase in its global prevalence since 1990 [56]. In African-American and Caucasian adults with CKD, there is an increase in the prevalence of AF. The prevalence increases as the CKD progresses and is highest in stages four or five [57]. The incidence of AF in CKD increases as the disease progresses [58]. Nelson et al. published a retrospective cohort study in 2012 using the medicare 5% database to evaluate the incidence of AF with advancing CKD and their mortality rates in elderly patients. The data estimated the two-year incidence of AF in CKD stages one and two as 12.2%, and 14.4% in stages three to five, 13.4% for unknown stage, compared to 7.5% in patients without CKD. The study concluded that the incidence of AF increases with the progression of CKD (Table 3) [59]. There exists a bidirectional relationship between CKD and AF. Renal impairment predisposes to the initiation and maintenance of AF, while unmanageable AF hastens the decline in renal function [60]. The common risk factors that are shared by CKD and AF are age, male sex, cardiovascular disease, hypertension, diabetes, heart failure, and obesity [47,57]. Watanabe et al. in 2009, conducted a prospective cohort study with 235818 general Japanese population to find out if a bidirectional relationship existed between CKD and AF. During a follow-up for 5.9 +/- 2.4 years, a total of 2947 members developed AF. They measured creatinine clearance and eGFR at baseline to assess the risk of AF and calculated the hazards ratio (HR) with 95% CI for eGFR 30 to 59 and <30 mL/min/1.73 m^2^ which were 1.32 (1.08-1.62) and 1.57 (0.89-2.77), respectively. On further follow-up, 7791 of them developed renal dysfunction, and 11307 developed proteinuria. The HR (95% CI) for the development of renal dysfunction in AF was 1.77 (1.50-2.10), and that of proteinuria in AF was 2.20 (1.92-2.52). At the completion of the study, they concluded that kidney dysfunction augmented the risk of new-onset AF, and the AF increased the risk of development of kidney disease (Table 3) [61]. CKD and AF both aid in blood clots formation by causing a prothrombotic state by affecting individual components of Virchow's triad. In worsening CKD patients with AF, stasis of blood occurs in the left atrium which can precipitate thrombus formation. patients with CKD due to endothelial dysfunction can also develop a thrombus. In the early stages of CKD, platelet activation can occur leading to thrombus formation. CKD patients have an increase in procoagulant and inflammatory markers which can increase the clotting tendency of the individual [16,47]. Piccini et al. conducted a cohort study which was published in 2013. They enrolled 13559 patients with non-valvular AF in the study and assessed the risk factors associated with stroke and systemic embolism occurrence by a risk score developed from the ROCKET AF trial. In the end, the study concluded that kidney function is a strong predictor of stroke and systemic embolism (Table 3) [62]. As CKD and AF both lead to a prothrombotic state, they independently can cause thromboembolic events manifesting as stroke. The risk of thromboembolism increases when both CKD and AF are present. The assessment of stroke risk in AF patients with CKD is done using the CHA_2_DS_2_VASc score (congestive heart failure, hypertension, age, diabetes, stroke, vascular disease, and sex), a score of three or more in females and two or more in males warrants oral anticoagulative therapy. Furthermore, a decline in renal function increases the risk of stroke in AF patients [47,63]. Guo et al. performed a cohort study which was published in 2013 to assess the risk of stroke in patients with AF and progressive renal dysfunction. They included 617 patients with AF and followed them up for two years. During which their renal function was assessed by estimating their eGFR at baseline, six months, and 12 months and looking for clinical events like ischemic stroke, major bleeding, and death. The study concluded that progressive renal dysfunction augmented the risk of stroke in patients with AF (Table 3) [64]. AF development in CKD patients causes an increase in mortality [65]. Airy et al. in 2018 published a study to assess the relationship between AF and cause-specific mortality in the CKD population. They included 62459 patients with eGFR 15-59 mL/min/1.73 m^2^ out of which 6639 had AF and 55820 did not have AF, during a median follow-up of 4.1 years a total of 19094 patients died and the cause of death of 18854 was known. AF had a 23% (95% CI 18-29%) higher risk of all-cause mortality, 45% (95% CI 31-61%) higher risk of cardiovascular mortality, and 13% (95% CI 3-22%) lower risk of malignancy-related mortality after multivariable adjustment. The study concluded that the presence of AF in the non-dialysis-dependent CKD population was associated with higher all-cause and cardiovascular mortality (Table 3) [66].

**Table 3:** Summarized data of studies to understand the clinical correlation between AF and CKD. CKD: chronic kidney disease; AF: atrial fibrillation; eGFR: estimated glomerular filtration rate; HR: hazards ratio; CI: confidence interval

Reference	Year	Design	Population	Methods/results	Conclusion
Nelson et al. [59]	2012	Retrospective cohort study	55962 patients using medicare 5% database	Calculating incidence rates of AF in different stages of CKD	As the stage of CKD increases, there is an increased incidence of AF
Watanabe et al. [61]	2009	Prospective cohort study	235818 general Japanese population	HR for AF in CKD patients with eGFR 30-50 and <30 mL/min/1.73 m^2^ are 1.32 (95% CI 1.08-1.62) and 1.57 (95% CI 0.89-2.77), respectively and HR for development of kidney dysfunction and proteinuria in AF patients are 1.77 (95% CI 1.50-2.10) and 2.20 (95% CI 1.92-2.52), respectively	Kidney dysfunction augments AF risk and AF increases the development of kidney disease
Piccini et al. [62]	2013	Cohort study	13559 individuals with non-valvular AF	A risk score developed from the ROCKET AF study	Kidney function is a strong predictor of stroke and systemic embolism
Guo et al. [64]	2013	Cohort study	617 patients with AF	Measuring eGFR at baseline, six and 12 months and looking for clinical events like stroke, major bleeding, and death	Progressive renal failure augments the risk of stroke in AF patients
Airy et al. [66]	2018		62459 patients with CKD	AF had a 23% (95% CI 18-29%) higher risk of all-cause mortality, 45% (95% CI 31-61%) higher risk of cardiovascular	The presence of AF in the non-dialysis dependent CKD population was associated with higher all-cause

Management of AF in CKD

The management of AF encompasses controlling rhythm or rate of the heart and systemic thromboembolism prevention.

Rate and Rhythm Control

Rate control: In the general population, rate control is preferred over rhythm control for both symptom improvement and stroke prevention. The downside of rhythm control drugs includes unwanted side effects, toxicity, difficulty to achieve maintenance of normal sinus rhythm with long-term use, prolonged hospitalization, and are more expensive compared to rate control drugs. However, there is no clear evidence to prove that rate control is superior to rhythm control in CKD patients [67]. Moderate rate control (resting heart rate <110 bpm) was associated with improved outcomes (hospitalization for heart failure, stroke, systemic embolism, bleeding, life-threatening arrhythmias, and death from cardiovascular causes) than intense rate control (resting heart rate <80 bpm and heart rate during moderate exercise 110 bpm), then again there is no proper evidence that suggests moderate rate control has better outcomes over intense control in CKD population [67]. The major drug classes used for rate control are beta-blockers, non-dihydropyridine calcium channel blockers (e.g., verapamil and diltiazem), and digoxin. Water-soluble drugs are to be avoided in CKD as they can accumulate in the body due to decreased renal excretion. As a result, water soluble beta-blockers like atenolol and sotalol are to be avoided. The dose of drugs like bisoprolol, which have a mixed metabolism (metabolized by the liver and kidney) is to be adjusted. Therefore, the best treatment option for CKD patients is to withhold hydrophilic drugs and prescribe lipophilic drugs, which are metabolized in the liver. The commonly used drugs are diltiazem and lipophilic beta-blockers like metoprolol and carvedilol [68]. The use of digoxin for rate control in the late stages of CKD is generally avoided because of its increased mortality risk as about 85% of the drug is renal excreted. It has a narrow therapeutic index, a long half-life (t 1/2), and it is arrhythmogenic as hypokalemia can occur during hemodialysis [67]. Yang et al. published a cohort study in 2021 to assess the risk of digoxin in CKD patients to treat AF and HF using the pre-end-stage renal disease care program registry and national health insurance database in Taiwan. A total of 31933 patients with CKD were taken into the study out of which 400 were labeled as digoxin users and 2200 were labeled as non-user group, they were matched with age and sex. Multivariable Cox proportional-hazards and sub-distribution hazards models were used to determine the HRs of the acute coronary syndrome, ischemic stroke, and rapid eGFR decline for digoxin use in CKD patients who had sub-distribution hazard ratio (sHR) of 1.18 (95% CI 0.75-1.86), sHR of 1.42 (95% CI 0.85-2.37), and sHR of 1.00 (95% CI 0.78-1.27), respectively. Results show that the all-cause mortality of the digoxin user group was higher compared to the non-user groups after adjusting the covariates (adjusted hazard ratio, aHR 1.63; 95% CI 1.23-2.17). The study concluded that digoxin use in later stages of CKD was associated with increased mortality [69].

Rhythm control: The rhythm control is done by using Vaughan Williams class IA (disopyramide, quinidine), class IC (flecainide, propafenone), and class III (amiodarone, dofetilide, dronedarone, sotalol) antiarrhythmic drugs in the general population [63]. The lipophilic drug propafenone has a low proarrhythmic potential and is a better choice in CKD patients [70]. Amiodarone can be used in CKD patients. However, because of its long-term effects like thyroid dysfunction (hyperthyroidism and hypothyroidism) and pulmonary toxicity, it has to be given with caution [71]. Dronedarone requires no initial dose adjustment as it is mostly excreted through feces. Having said that, it is contraindicated in type III/IV New York Heart Association (NYHA) heart failure and is less effective compared to amiodarone [72]. Ibutilide should be used cautiously in CKD patients because of its proarrhythmic potential in hypokalemia and hypomagnesemia states despite being a good agent for cardioversion of AF or atrial flutter in CKD [73]. Dofetilide needs dose adjustment in patients with CKD because of its increased risk of arrhythmias as it is mostly excreted by kidneys [74]. Flecainide is excreted by kidneys and is not recommended in CKD patients as there have been reports of toxicity in severe CKD [75].

Cardioversion and Cardiac Ablation

Cardioversion: Direct current cardioversion as a treatment choice is recommended in case of AF with a rapid ventricular response that doesn't respond to drug therapy and worsens the already present cardiovascular problems like hypotension and HF. It is also done in the case of patients with AF having hemodynamic instability [63]. Without maintenance antiarrhythmic therapy, the recurrence of AF after cardioversion is high and it depends on baseline renal function [67]. Schmidt et al. in 2010 published a study to assess the relationship between the level of impaired renal function and its influence on the maintenance of sinus rhythm after successful electric cardioversion. A total of 102 patients with persistent AF who underwent successful cardioversion were included in the study. They were followed up for AF recurrence through telephone interviews, Holter ECG and ECG sent by primary care providers. The patients were divided into four groups based on the GFR measured before and one month after cardioversion (I >90 mL/min/1.73 m^2^, II 60-90 mL/min/1.73 m^2^, III 30-59 mL/min/1.73 m^2^, and IV <30 mL/min/1.73 m^2^). The recurrence rate of AF was significantly higher in patients with severely or moderately decreased renal function. The study concluded that there is an increased risk of AF recurrence after successful electric cardioversion in patients with impaired renal function [76].

Cardiac ablation: Catheter ablation is done in the case of symptomatic paroxysmal AF refractory or intolerant to at least one class I or III antiarrhythmic medication when a rhythm control strategy is thought to be effective [63]. Cardiac ablation is also associated with increased rates of recurrence and it is less efficacious in CKD patients compared to the general population. Nonetheless, when performed successfully causes an improvement in renal function [67]. Navaravong et al. published a study in 2015 to assess the effect of AF ablation on kidney function in CKD patients. A total of 392 patients with CKD were included in the study. Out of which 118 had stage one CKD, 198 had stage two CKD, 56 had stage three A (3A) CKD, 20 had stage three B (3B) CKD, and patients with eGFR <30 mL/min/1.73 m^2^ were excluded from the study. After a median of 115 post-ablation, eGFR significantly increased in CKD stage two (74±9-80±23; p=0.04), three A (53±5-69±24; p<0.001), three B (40±4-71±28; p<0.01), and decreased in CKD stage one (109±18-82±28; p<0.001). The study concluded that there was a significant improvement in renal function in patients with CKD after a successful ablation [77].

Prevention of Stroke and Risk of Thromboembolism

Anticoagulants: There is an increased risk of thromboembolism in CKD patients with AF as a result of disorganized atrial contraction; paradoxically, there is an increased risk of bleeding as well in CKD patients compared to the general population [68]. The prevention of thromboembolic events in AF can be done with the administration of oral anticoagulant therapy or antiplatelet drugs. Oral anticoagulants are prescribed based on the CHA_2_DS_2_VASc score. A score of >3 in males and >2 in females warrants initiation of Warfarin which is a vitamin K antagonist (VKA) or direct oral anticoagulants (DOACs) like dabigatran, apixaban, and rivaroxaban [63]. Hypertension, abnormal renal/liver function, stroke, bleeding history or predisposition, labile INR, elderly, drugs/alcohol concomitantly (HAS-BLED) scoring system is used to identify patients who are at a higher risk of bleeding when on anticoagulation; one point each is given for hypertension, abnormal liver or renal function, stroke, history of bleeding, labile International Normalized Ratio (INR), and drugs. A score ≥3 is considered high risk, this system does not take into account mild-to-moderate renal dysfunction. This can have a significant impact on bleeding risks. It also defines renal dysfunction as ESRD with long-term hemodialysis therapy, history of renal transplantation, or serum creatinine ≥2.26 mg/dL [78].

There is data that says the use of anticoagulants in patients with AF and CKD decreases the incidence of thromboembolic events without increasing the bleeding risk [79]. Hart et al. conducted a meta-analysis which was published in 2007 to determine the safety and efficacy of antithrombotic agents in preventing stroke in AF patients. A total of 29 randomized control trials (RCTs) with 28044 participants were included in the study. They calculated the risk reduction (RR) between controls and patients given adjusted-dose warfarin 64% (95% CI, 49-74%) and between controls and patients given antiplatelet drugs 22% (95% CI, 6-35%). They also calculated the relative risk reduction (RRR) in patients given dose-adjusted warfarin and antiplatelet agents which were 39% (95% CI, 22-52%), indicating that adjusted-dose warfarin was more efficacious than antiplatelet drugs. At the end of the study, they concluded that dose-adjusted warfarin and antiplatelet agents reduce stroke by 60% and 20%, respectively, in AF patients [80]. There is evidence that suggests the use of warfarin increases the risk of bleeding in CKD patients [67]. Jun et al. performed a retrospective cohort study which was published in 2015, to assess the rates of major bleeding in older adults with AF on warfarin therapy by the level of kidney function. A total of 12403 adults aging 66 years or more with AF who were started on warfarin were included, and adults with ESRD were excluded. Both unadjusted and adjusted bleeding rates of warfarin therapy in AF patients with reduced eGFR were calculated in 1443 patients who experienced major bleeding during a median follow-up of 2.1 years, they were high after the first 30 days of treatment initiation. The adjusted bleeding rates per 100 person-years in patients with eGFR <15 mL/min/1.73 m^2^ and >90 mL/min/1.73 m^2^ were 63.4 (95% CI 24.9-161.6) and 6.1 (95% CI 1.9-19.4), respectively. The increase in major bleeding rates was mainly due to gastrointestinal (GI) bleeding, which was 3.5-fold greater in eGFR with <15 mL/min/1.73 m^2^ compared to eGFR >90 mL/min/1.73 m^2^. The study concluded that reduced kidney function was associated with an increased risk of major bleeding in adults with AF on warfarin, and the risk was greater in the first 30 days of starting warfarin [81]. Warfarin can cause warfarin-induced nephropathy through different mechanisms, and the risk is higher in CKD patients compared to patients with normal kidney function [82]. Patients on dialysis have a higher risk of stroke. Patients on dialysis who received anticoagulant and VKA medication did not see any reduction in strokes. Additionally, the chance of bleeding increased. The administration of warfarin in CKD patients with uremia can augment cardiovascular calcification and calciphylaxis [67].

DAOCs: DOACs are newer anticoagulants mainly composed of two classes, direct thrombin inhibitors (e.g., Dabigatran) and direct Xa inhibitors (e.g., rivaroxaban and apixaban). They are safe compared to VKAs because they have few drug interactions and rapid onset/offset, and they do not require monitoring of coagulation [63]. A study revealed that the use of dabigatran demonstrated superiority over warfarin for the prevention of stroke and systemic embolism [83]. ROCKET AF study compared rivaroxaban with warfarin for stroke and systemic embolization reduction. It showed that rivaroxaban was non-inferior to warfarin and had lower rates of major bleeding [84]. The ARISTOTLE trial came to a conclusion that apixaban is superior to warfarin in stroke and systemic embolization reduction with lower rates of bleeding and in different stages of CKD; both rivaroxaban and apixaban showed no difference in treatment effect heterogeneity [85,86]. The DAOCs are variably excreted by kidneys. As a result, they may accumulate in the body and have a prolonged effect. The reversal therapy is not as clear as it is for warfarin with vitamin K. The current advice for managing hemorrhage is by administering activated charcoal for reduction of absorption from the gut, tranexamic acid, fluids to increase renal perfusion and drug excretion, and finally performing hemodialysis [87,88].

Antiplatelets: The use of aspirin for stroke prevention in AF is quite common, but the recent guidelines suggest not to use aspirin for stroke prevention because of its inferiority to warfarin in the general population [63]. Vazquez et al. published a meta-analysis in 2015 to assess the benefits of low-intensity anticoagulation over Aspirin in stroke prevention and compare their outcomes in non-valvular AF patients. From the 6309 initially screened studies, only three were selected for the study. A total of 963 patients were present in the study, out of which, 460 were treated with low-intensity anticoagulation and 503 were given aspirin for stroke prevention. When compared with low-intensity anticoagulation, aspirin did not reduce stroke and systemic embolism incidence OR (95% CI 0.57-1.56). Furthermore, the incidence of major bleeding and vascular death had an OR of 1.06 (95% CI 0.43-2.62) and 1.04 (95% CI 0.61-1.75), respectively. There was a significant increase in all-cause mortality in patients who used aspirin OR (95% CI 1.12-2.48). In the end, the study concluded that low-intensity anticoagulation is more beneficial than aspirin in treating non-valvular AF and there is an increase in all-cause mortality with aspirin usage [89]. In patients with renal dysfunction, the antiplatelet effect of clopidogrel is not so effective and it can be due to the pre-occupation of gpIIb/IIIa receptor with uremic toxins and activation of inhibitory mechanisms of nitric oxide (NO) [68].

Practical aspects during treatment initiation to be considered

Before starting these medicines, patients must undergo a baseline renal function test. Monitoring has to be done annually or more frequently in high-risk patients because the renal function can deteriorate while on treatment. The renal function must be re-assessed in case of acute illnesses (infections, acute HF, etc.) because they transiently affect the kidney's function and proper care must be taken in prescribing other drugs which may affect the kidney [90].

To prevent patients with severe renal failure from over-anticoagulating, a start-low, go-slow strategy for warfarin dose is advocated. This is due to the fact that compared to the management of normal kidney function patients, individuals with severe renal failure need a much lower daily dosage of warfarin to achieve therapeutic INR. Which is most likely connected to the decreased cytochrome P450 activity in CKD. As a result, the INR has to be monitored frequently in patients on warfarin therapy [90].

The dosing of DOACs drugs is also determined by renal function. For instance, in patients with a creatinine clearance (CrCl) of <15 mL/min rivaroxaban is contraindicated, and in those with a CrCl of 15-49 mL/min, it is reduced from 20 mg to 15 mg daily. Apixaban is to be given twice daily with a dose of 2.5 mg in patients with CrCl 15-29 mL/min [90].

Limitations

The drawbacks of this study are that we used only PubMed as our database. As CKD leads to AF through multiple mechanisms, not all of them were briefly discussed and covered. The role of anticoagulants in the prevention of thromboembolic events in ESRD patients was not highlighted due to the lack of enough studies.

## Conclusions

On and after covering studies in this article, AF is a common arrhythmia in CKD patients which can manifest as a consequence of many conditions, a few of them being inflammation, anemia, imbalance in electrolytes, RAAS, neurohormonal system activation, and uremia. Keeping the above-mentioned facts in mind, we attempted to emphasize AF in patients with CKD in this review article to address the clinical impact of AF in CKD patients and its management. Due to the bidirectional relationship between AF and CKD, proper management can help prevent the deterioration of the existing CKD, avoid events like stroke, and ultimately reduce mortality. Medical practitioners should try to avoid drugs like (atenolol, sotalol, etc.) that are commonly used to treat AF in the general population, as patients with CKD have reduced renal function and cannot eliminate them, causing unwanted complications. We believe that this article will serve as a foundation and invoke interest in researchers to dig deep into this issue. We recommend researchers perform and publish more studies on this topic, especially in the ESRD population in the future.
